# Downregulation of Inflammatory Response via Nrf2/Trx1/TXNIP Axis in Oxidative Stress-Induced ARPE-19 Cells and Mouse Model of AMD

**DOI:** 10.1155/2022/1497813

**Published:** 2022-08-12

**Authors:** Qian Yang, Wenting Cai, Huizi Jin, Tianyi Shen, Jing Yu

**Affiliations:** Department of Ophthalmology, Shanghai Tenth People's Hospital, School of Medicine, Tongji University, Shanghai 200072, China

## Abstract

**Aim:**

Chronic inflammation is crucial for age-related macular degeneration (AMD) pathogenesis. However, the mechanism involved in activating inflammation remains unclear. This study is aimed at investigating whether nuclear factor erythrocyte-associated factor 2 (Nrf2) negatively regulated the Nod-like receptor protein 3 (NLRP3) inflammasomes through the thioredoxin 1 (Trx1)/thioredoxin interaction protein (TXNIP) complex.

**Methods:**

We determined the optimal hydrogen peroxide (H_2_O_2_) concentration, time, and changes in reactive oxygen species (ROS) levels. We also constructed animal models using blue LED irradiation. Then, the expression of Nrf2, TXNIP, Trx1, NLRP3, and inflammation-related factors and proteins, along with the changes in retinal thickness and functional status, was analyzed.

**Results:**

The oxidative stress model was established after 1 h intervention with 100 *μ*M H_2_O_2_. Nrf2 reduced ROS production, protected the ultrastructure of mitochondria, increased the thickness of the ONL layer, and increased the amplitude of a- and b-wave amplitudes in ERG. Trx1 knockdown increased the production of ROS, damaged the ultrastructure of mitochondria, reduced the thickness of the other ONL layer, and reduced the amplitudes of a- and b-waves in the electroretinogram (ERG). Thus, TXNIP in the cytoplasm activated the inflammasomes.

**Conclusions:**

Nrf2 showed antioxidant and anti-inflammatory activity in the H_2_O_2_-induced cell stress model and blue LED-induced retinal light damage model. TXNIP transferred from the nucleus to the cytoplasm, activated NLRP3, and aggravated the retinal injury in both the cell stress model and the animal blue LED model. In contrast, Trx1 knockout promoted this process. This study revealed the possible role of the thioredoxin system in developing AMD while also providing newer insights for the future treatment of AMD.

## 1. Introduction

Age-related macular degeneration (AMD) is mainly observed in the elderly, which leads to blurring and loss of vision [1]. Dry (nonexudative) and wet (neovascular) are the two forms of macular degeneration. Dry AMD is the disease of the retina with a neurodegenerative component. Drusen formation is one of the features in the early stages of AMD. Drusen is the extracellular deposit between the human retinal pigment epithelial cell (RPE) and Bruch's membrane. Moreover, it causes progressive loss of photoreceptors by degeneration and dysfunction of RPE as the disease progresses. Eventually, AMD may cause permanent blindness. Although the pathogenesis of AMD has been proposed continuously, the exact mechanism of this disease remains unclear. Previous studies have shown that inflammation is the major pathogenic pathway involved in AMD [2]. Nod-like receptor protein 3 (NLRP3) inflammasome is composed of various complexes associated with several diseases. Its activation can increase the release of inflammatory factors such as caspase-1, IL-1*β*, and IL-18, leading to the chronic persistent inflammatory response. These elevated levels cause RPE damage and further aggravate AMD [3, 4]. However, the mechanism of activation of inflammasomes in AMD remains unclear.

Our previous study showed that miR-27a expression was increased in the blood of patients with AMD. Importantly, thioredoxin interaction protein (TXNIP) was identified as a downstream molecule of miR-27a [5]. TXNIP performs many functions, such as mediating oxidative stress and inhibiting cell proliferation [6]. Thioredoxin 1 (Trx1) is an endogenous TXNIP inhibitor [7]. Trx1 is a ubiquitous small molecule with various biological activities and is known to be upregulated in multiple oxidative stress responses [8]. In addition, it is known to cause RPE dysfunction in retinal neurodegenerative diseases [9, 10].

The nuclear factor erythrocyte-associated factor 2 (Nrf2) is a central regulator of REDOX homeostasis and induces Trx1 expression in cerebral neuropathy [11, 12]. Furthermore, recent studies have shown that increasing the level of Nrf2 can reduce the content of NLRP3 inflammasomes in ARPE-19 [13]. Thus, we explored the specific mechanism of NLRP3 activation in AMD and verified whether Nrf2 negatively regulated NLRP3 by regulating the Trx1/TXNIP complex.

## 2. Materials and Methods

### 2.1. Reagents

Human retinal pigment epithelium cell lines (ARPE-19) were purchased from the Academy of Sciences (Shanghai, China). Fetal bovine serum (10%, Gibco, USA) in DMEM/F12 cell medium (Hyclone, USA) with penicillin/streptomycin was used for cell culture. Cell counting kit- (CCK-) 8 and the Reactive Oxygen Species Assay Kit were purchased from Yeasen (Shanghai, China). tBHQ (30 *μ*M) dissolved in DMSO was purchased from Topscience (Shanghai, China). Primary antibodies for Nrf2, IL-1*β*, IL-18, and caspase-1 were purchased from Abcam (Abcam Inc., UK). TXNIP and Trx1 were procured from Proteintech (Proteintech, USA). *β*-Actin and lamin B1 antibodies were purchased from CST (CST, USA); goat anti-rabbit IgG antibody (1 : 5000) from ABclonal Technology, Wuhan, China; goat anti-Mouse IgG antibody (1 : 2000) from Sigma-Aldrich. Trx1 and control siRNA were purchased from RiboBio (Guangzhou, China). C57BL/6 mice were obtained from Beijing Vital River Laboratory Animal Technology Co., Ltd. (Beijing, China).

### 2.2. Cell Culture

ARPE-19 cells were cultured in DMEM/F12 medium in an incubator at 37°C with 5% CO_2_. The cells were passaged at approximately 80% confluency to keep the cells in a good state of growth.

### 2.3. RNA Silencing

At 30-50% confluency, cells were transfected with Trx1 and Ctrl siRNA using a customized siRNA reagent system. The medium was changed after 4 h. RNA was extracted at 48 h, and protein was extracted at 72 h for subsequent experiments.

### 2.4. Cell Viability Assay

ARPE-19 cells at the logarithmic growth stage were treated with 0, 100, 200, 300, 400, and 500 *μ*mol/L of H_2_O_2_ (Suicheng, Henan, China) for 1 h, 1.5 h, and 2 h. After washing with PBS, 110 *μ*L of the liquid mixture (100 *μ*L basic medium and 10 *μ*L CCK-8 solution) was added to each well, followed by the addition of PBS solution in the periphery of the well to eliminate interference. After incubating for 4 hours in a 37°C incubator, optical density was measured at 450 nm under the enzyme label analyzer (Synergy H4; BioTek, Winooski, VT, USA). Experiments were performed in triplicates in three independent settings.

### 2.5. Measurement of ROS

ARPE-19 cells were treated with H_2_O_2_ (0, 100, 200, 300, 400, and 500 *μ*mol/L) for 1 h. Based on the results from CCK-8 and flow cytometry assays, 100 *μ*mol/L H_2_O_2_ was used for 1 h for the subsequent experiences. Cells were pretreated with 30 *μ*M tBHQ for 12 h to determine the role of Nrf2 and Trx1. According to the above treatment, the cells were divided into the following groups: (A) blank control; (B) 100 *μ*M H_2_O_2_; (C) tBHQ; (D) tBHQ+H_2_O_2_; (E) tBHQ+siTrx1+H_2_O_2_. Next, 1 *μ*L of DCFH-DA fluorescent probe was added to the 6-well plate and incubated for 30 min. The cells were collected after washing with PBS. The ROS generation was recorded using a FACSCanto II flow cytometer (BD). The results were presented as the average of three independent assays.

### 2.6. Immunofluorescence Staining (IF)

ARPE-19 cells were seeded 4 × 10^4^/well in 24-well plates. The cells were treated with 100 *μ*M H_2_O_2_ for 1 h. Subsequently, the cells were fixed with 4% cold paraformaldehyde for 20 min. Then, the cells were washed thrice with PBS, and 200 *μ*L liquid (5% BSA+0.2% Triton) was added. The cells were incubated for 1 h. Then, the cells were incubated overnight with an anti-TXNIP antibody (1 : 200) at 4°C. Next, cells were incubated with fluorescent-labeled secondary anti-rabbit IgG (1 : 500) for 1 h at room temperature and stained with 1 *μ*g/mL DAPI for 10 min. After drying, the slides were imaged using a fluorescence microscope (Leica Solms, Germany).

Meanwhile, for *in vitro* experiments, cells were divided into five groups: (A) control; (B) H_2_O_2_; (C) tBHQ; (D) tBHQ+H_2_O_2_; (E) tBHQ+siTrx1+H_2_O_2_. A 200 *μ*L liquid (5% BSA+0.2% Triton) was added into the cells and incubated for 1 h. Then, anti-IL-18 and anti-IL-1*β* were added, and the cells were incubated overnight. A secondary antibody was added and incubated for another 1 h. At last, the slices were imaged on a fluorescence microscope.

### 2.7. Cell Scratch Wound Assay

1 × 10^5^ cells/well were seeded in a 6-well plate in the following groups: (A) control; (B) H_2_O_2_; (C) tBHQ; (D) tBHQ+H_2_O_2_; (E) tBHQ+siTrx1+H_2_O_2_. We drew three balanced lines at the bottom of the 6-well plate. When the cell fusion degree reached 80%, a 200 *μ*L pipette tip was used to scratch perpendicular to the line on the back of the plate hole. The scratched cells were washed with PBS. The media was replaced with a fresh basal medium. The cells were cultured at 37°C in a 5% CO_2_ cell incubator. Images were recorded at 0 h, 24 h, and 48 h using a microscope and photographed. The mobility was calculated according to the formula (distance/scratch width). We set repeat holes in each group, and the experiments were repeated thrice.

### 2.8. Transwell Assay

5 × 10^4^ cells/well were seeded in 200 *μ*L serum-free DMEM/F-12 culture medium in the upper chamber (8 mm pore size; Corning Incorporated, Corning, NY, USA) in the following groups: (A) control; (B) H_2_O_2_; (C) tBHQ; (D) tBHQ+H_2_O_2_; (E) tBHQ+siTrx1+H_2_O_2_. 500 *μ*L complete medium was added to the lower chamber. After 24 hours, the lower chamber membrane was cleaned with PBS, placed on a 24-well plate, fixed with 95% ethanol, and stained with 1% crystal violet. After drying, the 6-well plate was imaged using a microscope (Leica Solms, Germany).

### 2.9. Transmission Electron Microscopy (TEM)

The five groups of cells were treated as mentioned above and cleaned with PBS. The cells were immersed overnight in glutaraldehyde at 4°C, fixed at 4°C for 2 h with 1% osmium tetroxide. After dehydration, the cell masses were encased in resin, and slices were cut. The ARPE-19 cell's ultrastructure was visualized with electron microscopy. Besides, the sampling process was the same as in the previous study. The cells were divided into the following groups: (A) blank control; (B) H_2_O_2_; (C) tBHQ; (D) tBHQ+H_2_O_2_; (E) tBHQ+siTrx1+H_2_O_2_.

### 2.10. qRT-PCR

Total RNA was isolated using the EZ-press RNA Purification Kit (EZBioscience, USA). Cytoplasmic and nuclear RNA was extracted by the Cytoplasmic and Nuclear RNA Purification Kit (Norgen Biotek, Canada). The concentration and quality of the extracted RNA were determined by NanoDrop 2000c (Thermo Fisher Scientific, MA, USA). The cDNA was obtained using the PrimerScript RT reagent Kit (TaKaRa Bio Inc., Osaka, Japan). The relative expression of each gene was determined using the CFX384™ real-time system (Bio-Rad Laboratories, CA, USA).  Table 1 lists the PCR primers.

### 2.11. Western Blot Assay

The total protein of cells was extracted from the 6-well plate and washed using PBS. First, the total protein content was extracted by mixing RIPA lysis buffer (Beyotime, Shanghai, China) from the mouse retina. Next, the nuclear and cytoplasmic proteins were extracted using respective cytoplasmic and nuclear extraction kits (Yeasen, Shanghai, China). After that, the protein was quantified by BCA assay (Beyotime Institute of Biotechnology, Haimen, China). Next, the SDS-PAGE bands were transferred to the nitrocellulose membrane. Then, it was incubated at room temperature with 5% skim milk for 1 h. After washing with PBS, the membrane was incubated with primary antibodies, including anti-Nrf2, anti-NLRP3 inflammasome, anti-Trx1, anti-TXNIP, anti-caspase-1, anti-IL-1*β*, anti-IL-18, anti-*β*-actin, and anti-lamin B1 (1 : 1000, respectively) overnight at 4°C. Then, the membrane was incubated with the secondary antibody at room temperature for 2 h in the dark. The following secondary antibodies were used: goat anti-rabbit IgG (H+L) antibody and goat anti-mouse IgG (H+L) antibody (1 : 1000, respectively). Finally, the protein content was detected by Odyssey two-color infrared laser imaging system (LI-COR Biosciences, Lincoln, NE, USA).

### 2.12. Animal Care and Use

All animal experiments were performed based on the Association for Research in Vision and Ophthalmology Statement guidelines. Animal care and use were followed as per the Guidelines of Laboratory Animals published by the US National Institutes of Health. This study protocol was approved by Shanghai Tenth People's Hospital. 150 adult male C57BL/6 mice (4-6 weeks old) were used for the *in vivo* study. The mice were kept *ad libitum* in a 12 : 12 h light : dark cycle at a room temperature of 24°C.

### 2.13. Intravitreal Administration in Mice

The mice were anesthetized with an intraperitoneal injection of 3% sodium pentobarbital (1 mg/kg). Topical 0.5% promedocaine hydrochloride was used for the ocular surface anesthesia. The mice were intravitreally injected (IVI) with 2 *μ*L of either tBHQ (30 *μ*M), 2 *μ*L of Trx1 lentivirus (2 × 10^9^ TU/mL), or 2 *μ*L of saline as the control.

### 2.14. Animal Model

Mice were randomly divided into seven groups: control, Blue LED, DMSO+Blue LED, tBHQ+Blue LED, shCtrl+Blue LED, shTrx1+Blue LED, and tBHQ+shTrx1+Blue LED. TBHQ and shTrx1 were injected into the vitreous cavity at 12 h and 8 h before Blue LED irradiation. One drop of 0.5% tropicamide was applied topically to enable the dilation of the pupil before exposure to a Blue LED. A Blue LED with a wavelength of 460 nm and an intensity of 3000 lux was used for 2 h irradiation at a distance of 10 cm from the mouse eyes. After Blue LED exposure, the mice were kept in the dark environment for 24 h. Then, the light/dark cycle was resumed for 12 h.

### 2.15. ERG Recordings

All images were taken after 5 days of blue light exposure. Before recording the image, the mice were dark-adapted for 12 h. Next, pentobarbital sodium was injected intraperitoneally, and thiazide (6 mg/kg) was injected intramuscularly for deep anesthesia. All steps were recorded under red light in the darkroom, and the animal's body temperature was maintained at approximately 38°C. The anesthetized mice were attached to the platform. The corneal, the reference, and the ground electrode were placed on the cornea and inserted subcutaneously at the top and tail, respectively. Using an LED lamp, a single flash of light (3000 cd/m^2^ for 10 ms) was applied to the eye through the corneal electrode. The images were captured, and the peaks of a-waves and b-waves were calculated.

### 2.16. Hematoxylin and Eosin Staining

The mouse eyeball was fixed, and the retinal tissue was cut into 5 *μ*m sections. The sections were dewaxed with xylene, dehydrated with gradient ethanol (100%, 95%, 85%, and 75% ethanol) for 2 h, and then in tissue fixation for 24 h. The tissues were stained for 5 min with hematoxylin, differentiated with 1% HCl alcohol, stained with eosin, and dehydrated with gradient alcohol. After the xylene became transparent, neutral gum was used to seal the film. The mounted slides were photographed by optical microscope (Leica Microsystems, Wetzlar, Germany).

### 2.17. Immunohistochemical Staining

Retinal tissue sections were dewaxed with gradient ethanol. After antigen retrieval, it was treated with H_2_O_2_, PBS, and 5% BSA. The primary antibody was mixed with tissue and coincubated overnight in a wet box at 4°C. After washing with PBS, the second antibody (1 : 2000, A0277; Beyotime Biotechnology) was added and incubated at room temperature. The horseradish peroxidase-labeled avidin (A0303; Beyotime) was added at 37°C and incubated for 30 min. The sections were stained, sealed, and photographed under the optical microscope.

### 2.18. Statistical Analysis

The results were analyzed using SPSS 19.0 (IBM Corp., Armonk, NY, USA). Data were presented as mean ± SD from three independent experiments. The *t*-tests were used to compare two groups or one-way ANOVA to compare multiple groups. The LSD *t*-test was used to analyze the date of two group parameters. *P* values < 0.05 were considered statistically significant.

## 3. Result

### 3.1. Effect of H_2_O_2_ on ARPE-19 Cell Activity and ROS Production

**Figure 1:**
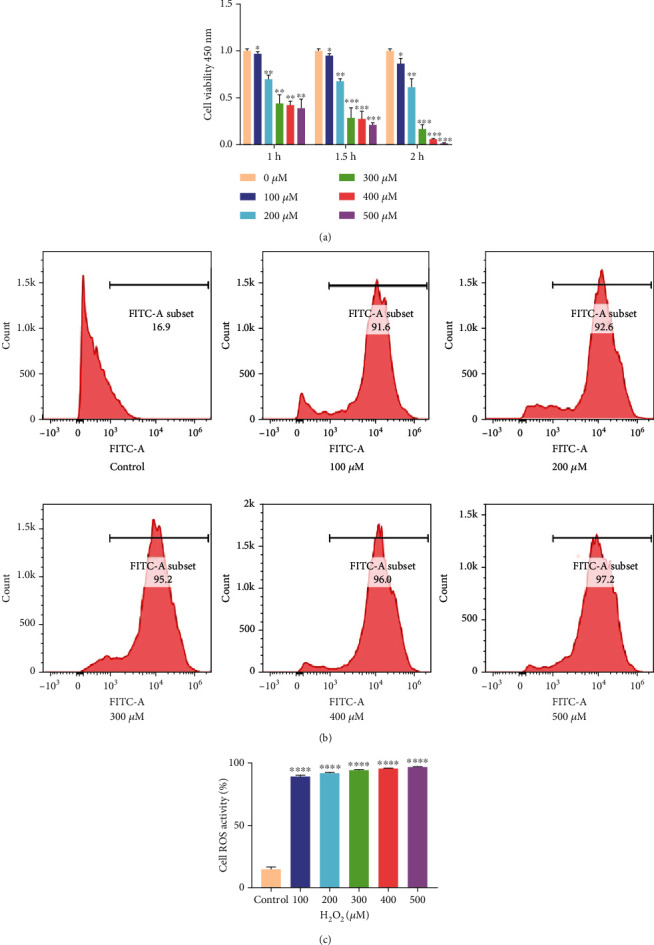
Effect of H_2_O_2_ on ARPE-19 cell activity and ROS production. (a) The statistical analysis of cell activity. (b) ROS production after treatment with different doses of H_2_O_2_. (c) Quantitative expression of cellular ROS. The data are shown as mean ± SD; *n* = 3. ^∗^*P* < 0.05; ^∗∗^*P* < 0.01; ^∗∗∗^*P* < 0.001; ^∗∗∗∗^*P* < 0.0001.

The ARPE-19 cells were exposed to various concentrations of H_2_O_2_ (0, 100, 200, 300, 400, and 500 *μ*mol/L) for 1 h,1.5 h, and 2 h to build a cell model of oxidative stress (Figure 1(a)). The cell activity was determined using the CCK-8 assay. At constant time, the cell activity decreased gradually with an increase in H_2_O_2_ concentration, and the cell activity became half at 300 *μ*M concentration. When cells were treated with 100 *μ*M H_2_O_2_ for 1 h, the cell activity decreased for the first time. When the treatment time was >1 h, the cell activity decreased significantly, which was not conducive to the subsequent experiments. At the same time, ARPE-19 cells treated with different concentrations of H_2_O_2_ for 1 h showed greater intracellular ROS generation than nontreated cells. With an increase in H_2_O_2_ concentration, no statistically significant difference was observed in ROS production among all groups (*P* > 0.05) (Figures 1(b) and 1(c)). Thus, this concentration (100 *μ*M H_2_O_2_ for 1 h) was selected for the experiments.

### 3.2. The Role of Nrf2 in the Inflammatory Activation of ARPE-19 Cells

**Figure 2:**
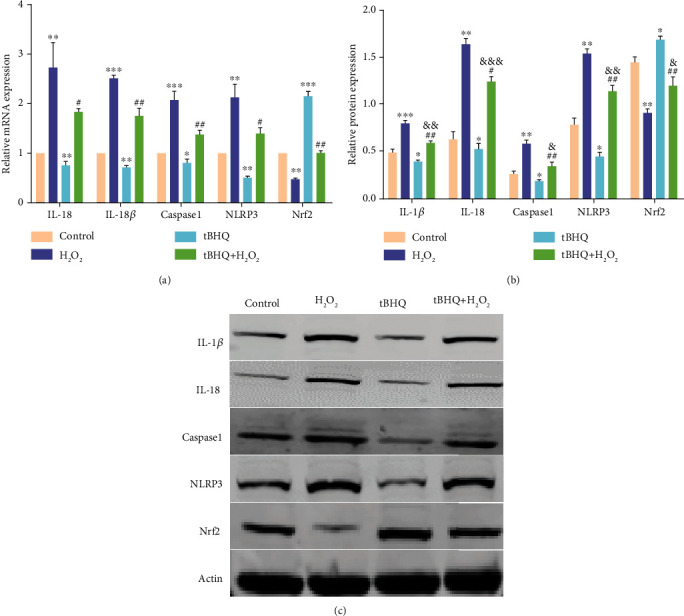
Effect of H_2_O_2_ on Nrf2, NLRP3, and related genes and proteins in ARPE-19 cells. (a) RT-PCR analysis of mRNA in the following four groups: control, H_2_O_2_, tBHQ, and tBHQ+H_2_O_2_. (b) Western blot of Nrf2, NLRP3, and related proteins in the following four groups: control, H_2_O_2_, tBHQ, and tBHQ+H_2_O_2_. (c) Quantification of Nrf2, NLRP3, and related protein expression. The data are shown as mean ± SD; *n* = 3. ^∗^*P* < 0.05 vs. control; ^∗∗^*P* < 0.01 vs. control; ^∗∗∗^*P* < 0.001 vs. control; ^#^*P* < 0.05 vs. H_2_O_2_; ^##^*P* < 0.01 vs. H_2_O_2_; ^&^*P* < 0.05 vs. tBHQ; ^&&^*P* < 0.01 vs. tBHQ; ^&&&^*P* < 0.001 vs. tBHQ.

We investigated whether we could activate Nrf2 through the tBHQ activator and assess its relationship with NLRP3 in oxidative stress. The results of qRT-PCR (Figure 2(a)) and western blot (Figures 2(b) and 2(c)) showed that NLRP3 inflammasome and the downstream factor (caspase-1, IL-1*β*, and IL-18) expression levels increased with a decrease in the Nrf2 expression after H_2_O_2_ treatment. Furthermore, compared with the other groups, Nrf2 expression increased, whereas NLRP3 and its downstream inflammatory factor expression decreased in the tBHQ group. Meanwhile, compared with the H_2_O_2_ group, the expression of Nrf2 increased, while the NLRP3 inflammasome and its downstream inflammatory factors significantly decreased in the tBHQ+H_2_O_2_ group. These results indicate that Nrf2 could inhibit inflammation and reduce oxidative stress-induced damage.

### 3.3. The Role of Trx1 in the Inflammatory Activation of ARPE-19 Cells

**Figure 3:**
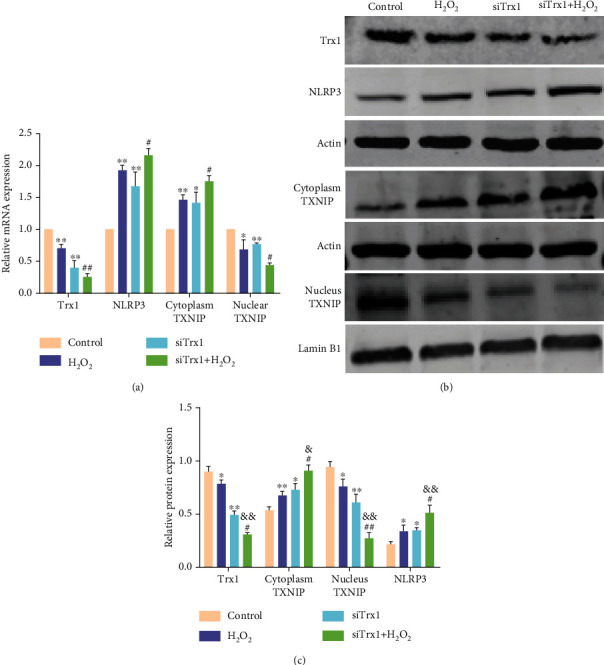
The role of Trx1 in the inflammatory activation of ARPE-19 cells. (a) RT-PCR analysis of mRNA in the following four groups: control, H_2_O_2_, siTrx1, and siTrx1+H_2_O_2_. (b) Western blot of Trx1, NLRP3, and TXNIP proteins in the following four groups: control, H_2_O_2_, siTrx1, and siTrx1+H_2_O_2_. (c) Quantification of Trx1, NLRP3, and TXNIP protein expression. The data are shown as mean ± SD; *n* = 3. ^∗^*P* < 0.05 vs. control; ^∗∗^*P* < 0.01 vs. control; ^#^*P* < 0.05 vs. H_2_O_2_; ^##^*P* < 0.01 vs. H_2_O_2_; ^&^*P* < 0.05 vs. tBHQ; ^&&^*P* < 0.01 vs. tBHQ.

We verified the relationship between Trx1 and TXNIP with NLRP3 inflammasome in oxidative stress at mRNA (Figure 3(a)) and protein (Figures 3(b) and 3(c)) levels. Compared with the control group, nuclear TXNIP and Trx1 expression was decreased in the H_2_O_2_ group, while the cytoplasmic TXNIP and NLRP3 inflammasome expression increased. Furthermore, in the siTrx1 groups, TXNIP expression was decreased in the nucleus and increased in NLRP3 and the cytoplasm. Besides, compared with the H_2_O_2_ group, nuclear TXNIP expression was decreased, whereas NLRP3 inflammasome and cytoplasm TXNIP expression was increased in the siTrx1+H_2_O_2_ group. Furthermore, immunofluorescence staining detected the changed subcellular localization of TXNIP in the H_2_O_2_-treated ARPE-19 cells. H_2_O_2_ promoted the transfer of TXNIP from the nucleus into the cytoplasm. These results indicated disintegration of the TXNIP and Trx1 complexes. The complexes were translocated into the cytoplasm after H_2_O_2_ treatment, leading to decreased nuclear and increased cytoplasmic expression of TXNIP and thus increased NLRP3 expression. Meanwhile, Trx1 knockdown promoted the TXNIP metastasis and enhanced the expression of NLRP3.

### 3.4. Nrf2 Inhibits NLRP3 in ARPE-19 Cells via Trx1/TXNIP Complex

**Figure 4:**
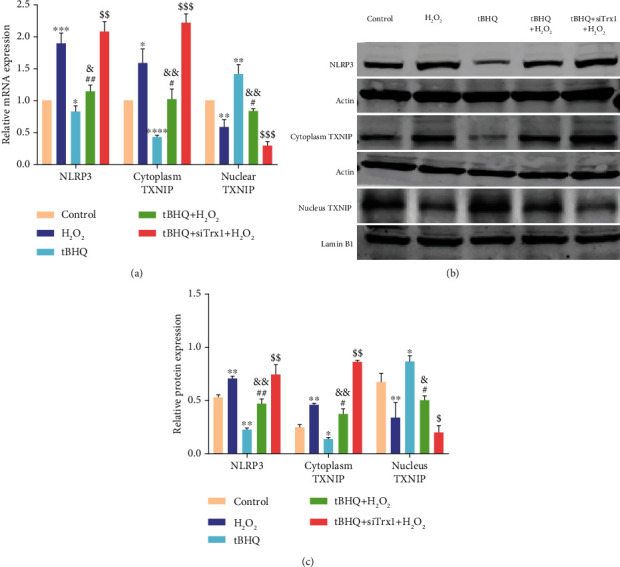
Effect of Nrf2 on the expression of TXNIP in ARPE-19 cell. (a) RT-PCR analysis of mRNA in the following four groups: control, H_2_O_2_, tBHQ, tBHQ+H_2_O_2_, and tBHQ+siTrx1+H_2_O_2_. (b) Western blot of NLRP3 and TXNIP proteins in the following five groups: control, H_2_O_2_, tBHQ, tBHQ+H_2_O_2_, and tBHQ+siTrx+H_2_O_2_. (c) Quantification of NLRP3 and TXNIP protein expression. The data are shown as mean ± SD; *n* = 3. ^∗^*P* < 0.05 vs. control; ^∗∗^*P* < 0.01 vs. control; ^∗∗∗^*P* < 0.001 vs. control; ^#^*P* < 0.05 vs. H_2_O_2_; ^##^*P* < 0.01 vs. H_2_O_2_; ^&^*P* < 0.05 vs. tBHQ; ^&&^*P* < 0.01 vs. tBHQ; ^$^*P* < 0.05 vs. tBHQ+H_2_O_2_; ^$$^*P* < 0.01 vs. tBHQ+H_2_O_2_; ^$$$^*P* < 0.001 vs. tBHQ+H_2_O_2_.

Based on mRNA (Figure 4(a)) and protein (Figures 4(b) and 4(c)) levels in the nucleus, the expression of TXNIP in the H_2_O_2_ group was decreased, while it was increased after treatment of tBHQ than the control group. Additionally, the tBHQ+siTrx1+H_2_O_2_ group showed a further decrease in TXNIP expression than the tBHQ+H_2_O_2_ group. However, the cytoplastic TXNIP expression in the H_2_O_2_ group was increased, while the cytoplastic TXNIP expression was decreased in the tBHQ group than in the control group. The tBHQ+siTrx1+H_2_O_2_ group had increased cytoplastic TXNIP expression compared with the tBHQ+H_2_O_2_ group. The changes in NLRP3 inflammasome were synchronized with the changes of TXNIP in the cytoplasm. The results indicated that nucleoplasmic metastasis occurred in TXNIP after H_2_O_2_ treatment, which was negatively regulated by Nrf2, with the involvement of Trx1.

### 3.5. Immunofluorescence Analysis of TXNIP, IL-18, and IL-1*β* in ARPE-19 Cells

**Figure 5:**
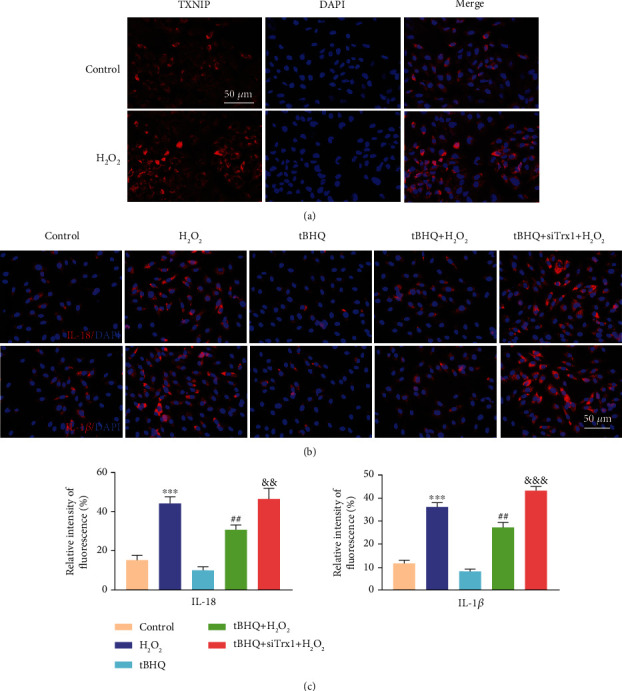
Immunofluorescence analysis of TXNIP, IL-18, and IL-1*β* in ARPE-19 cells. (a) Immunofluorescence analysis of TXNIP protein after the treatment of ARPE-19 cells with H_2_O_2_. (b) Immunofluorescence analysis of IL-18 and IL-1*β* protein in the following five groups: control, H_2_O_2_, tBHQ, tBHQ+H_2_O_2_, and tBHQ+siTrx+H_2_O_2_. (c) Quantification of relative fluorescence intensity about IL-18 and IL-1*β* proteins. Scale bar: 50 *μ*m. The data are shown as mean ± SD; *n* = 3. ^∗∗^*P* < 0.01 vs. control; ^∗∗∗^*P* < 0.001 vs. control; ^##^*P* < 0.01 vs. H_2_O_2_; ^&&^*P* < 0.01 vs. tBHQ+H_2_O_2_; ^&&&^*P* < 0.001 vs. tBHQ+H_2_O_2_.

H_2_O_2_-induced cytoplasmic TXNIP expression (Figure 5(a)) contrasted with extremely weak expression of unstimulated ARPE-19 cells. Meanwhile, to detect the oxidative damage caused by the Nrf2 and Trx1, the production of IL-18 and IL-1*β* was analyzed (Figure 5(b)). In the APRE-19 cell treated with H_2_O_2_, the production of IL-18 and IL-1*β* was significantly increased compared to control groups (*P* < 0.05) (Figure 5(c)). Meanwhile, Nrf2 was activated by tBHQ, weakening the H_2_O_2_-induced cell inflammation more than the H_2_O_2_ group. The production of IL-18 and IL-1*β* by the tBHQ group treated with si-Trx1 cells was higher than that by the tBHQ+H_2_O_2_ group (*P* < 0.05). These results suggested that Nrf2 and Trx1 significantly inhibited the H_2_O_2_-induced cell inflammation.

### 3.6. Effect of Nrf2 and Trx1 on Cell Migration

**Figure 6:**
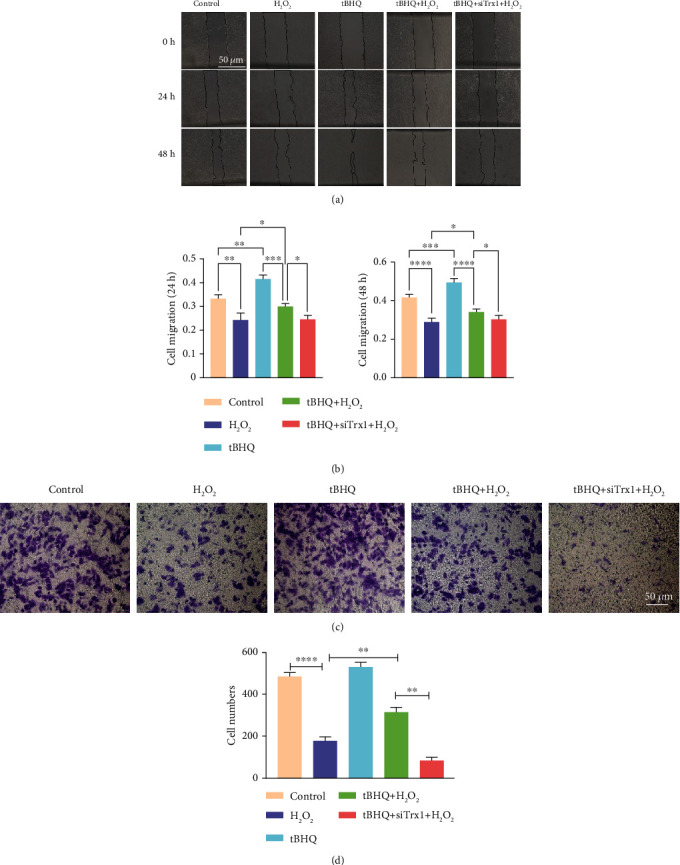
Effect of Nrf2 and Trx1 on cell migration. (a) A scratch test on ARPE-19 cells. The images were recorded at 0, 24, and 48 h. Magnification: ×500, scale bar: 50 *μ*m. (b) Quantitative analysis of the changes in cell migration at 24 and 48 h. (c) Transwell experiment after the treatment of ARPE-19 cells. (d) Quantitative analysis of invasion ability. Scale bar: 50 *μ*m. The data are shown as mean ± SD; *n* = 3. ^∗^*P* < 0.05, ^∗∗^*P* < 0.01, ^∗∗∗^*P* < 0.001, and ^∗∗∗∗^*P* < 0.0001.

The effect of Nrf2 and Trx1 on the function of ARPE-19 was assessed by scratch wound assay (Figures 6(a) and 6(b)) and transwell assay (Figures 6(c) and 6(d)). The scratch wound assay was employed to evaluate the cell migration. The scratch width was measured at 0 h, 24 h, and 48 h, and the relative mobility was calculated. Cell migration in the H_2_O_2_ group was significantly reduced compared with the control group (*P* < 0.05). Migration by the tBHQ group treated with si-Trx1 cells was lower than that of cells in the tBHQ+H_2_O_2_ group at 24 h and 48 h (*P* < 0.05). At the same time, Nrf2 was activated by tBHQ, intensifying the cell migration in the H_2_O_2_ group than the control group (*P* < 0.05). The transwell experiment was used to further validate the scratch test results. The transwell assay findings were similar to those of the scratch test. The H_2_O_2_ group that migrated through the filter was lower than the control group (*P* < 0.05). In the siTrx1+tBHQ+H_2_O_2_ group, cell migration was markedly decreased in the tBHQ+H_2_O_2_ group (*P* < 0.05). These results showed that H_2_O_2_ significantly inhibited ARPE-19 cell migration. The Nrf2 activation promoted the migration, while Trx1 knockdown further inhibited it.

### 3.7. Effect of Nrf2 and Trx1 on ROS Production and Mitochondrial Injury in Cells

**Figure 7:**
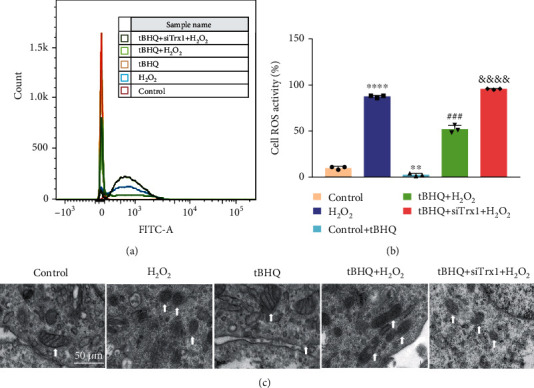
Effect of Nrf2 and Trx1 on ROS production and mitochondrial injury in cells. (a) ROS production in the following five groups: control, H_2_O_2_, tBHQ, tBHQ+H_2_O_2_, and tBHQ+siTrx+H_2_O_2_. (b) Quantitative expression of cellular ROS. (c) The change of mitochondrial morphology in the five groups. The arrow points to the mitochondria. Scale bar: 50 *μ*m. The data are shown as mean ± SD; *n* = 3. ^∗∗^*P* < 0.01 vs. control; ^∗∗∗∗^*P* < 0.0001 vs. control; ^###^*P* < 0.001 vs. H_2_O_2_; ^&&&&^*P* < 0.0001 vs. tBHQ+H_2_O_2_.

Flow cytometry was used to detect the production of cellular ROS. H_2_O_2_ stimulated the increase of ROS, whereas Nrf2 reduced the ROS production induced by H_2_O_2_ (Figures 7(a) and 7(b)). Meanwhile, ROS production was higher in the tBHQ+siTrx1+H_2_O_2_ group than in the other groups. Conversely, Nrf2 reduced the production of ROS, and the decreased Trx1 led to an increase in ROS production. Of note, both of them had an antioxidant effect.

To test the change of mitochondrial morphology in the five groups, TEM (Figure 7(c)) was used. The mitochondria and mitochondrial ridges were normal in the control and tBHQ groups. However, mitochondria became swollen, and mitochondrial ridges vanished in the H_2_O_2_ group, revealing damage to mitochondria caused by H_2_O_2_. Meanwhile, in the tBHQ+H_2_O_2_ groups, the mitochondrial swelling and ridge loss were more severe than in the tBHQ groups. Still, the swollen mitochondria with disorganized cristae were alleviated more than the H_2_O_2_ group. On the other hand, mitochondrial derangements and swelling were further aggravated in the tBHQ+siTrx1+H_2_O_2_ than the tBHQ+H_2_O_2_ group.

### 3.8. The Role of Nrf2 in the Inflammatory Activation in the Retina

**Figure 8:**
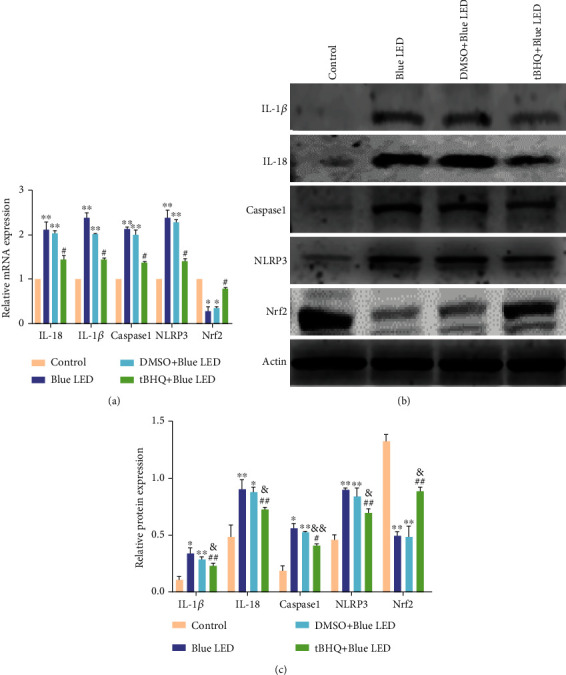
The role of Nrf2 in the inflammatory activation of retina. (a) RT-PCR analysis of mRNA in the following four groups: control, Blue LED, DMSO+Blue LED, and tBHQ+Blue LED. (b) Western blot of Nrf2, NLRP3, and related proteins in the following four groups: control, Blue LED, DMSO+Blue LED, and tBHQ+Blue LED. (c) Quantification of Nrf2, NLRP3, and related protein expression. Data represent mean ± SD; *n* = 3 for each group. ^∗^*P* < 0.05 vs. control; ^∗∗^*P* < 0.01 vs. control; ^#^*P* < 0.05 vs. Blue LED; ^##^*P* < 0.01 vs. Blue LED; ^&^*P* < 0.05 vs. tBHQ; ^&&^*P* < 0.01 vs. tBHQ.

The effects of Nrf2 on NLRP3 and its downstream factors were evaluated at the mRNA (Figure 8(a)) and protein (Figures 8(b) and 8(c)) levels. Nrf2 levels were significantly lower in the Blue LED exposure group than in the nonirradiated group. Compared with that in the Blue LED group, the Nrf2 expression in the tBHQ+Blue LED group was significantly increased. However, the expression of NLRP3 inflammasome and its downstream factors was significantly elevated in the Blue LED than in the control group. On the contrary, the expression of NLRP3 inflammasome and its downstream factors were decreased in the tBHQ+Blue LED group compared with the Blue LED and Blue LED+DMSO group. The results suggested that Nrf2 had a protective effect on the retina.

### 3.9. The Role of Trx1 in the Inflammatory Activation of the Retina

**Figure 9:**
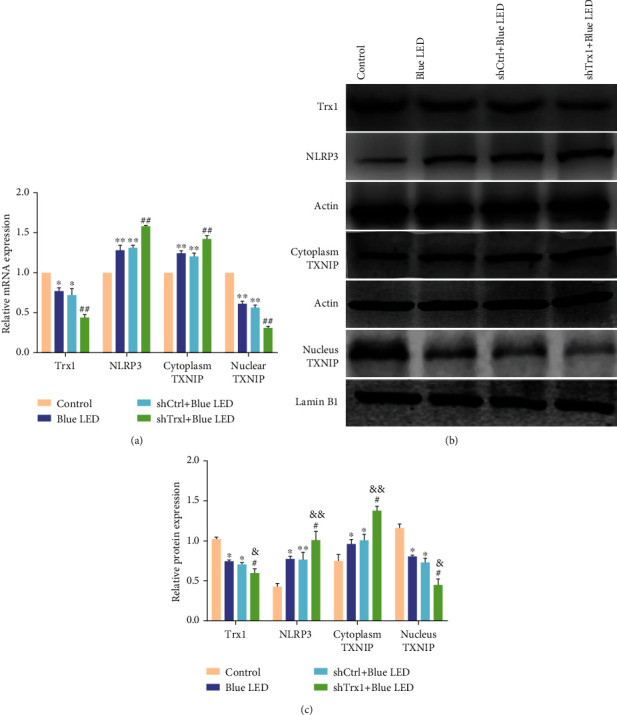
The role of Trx1 in the inflammatory activation of retina. (a) RT-PCR analysis of mRNA in the following four groups: control, Blue LED, shCtrl+Blue LED, and shTrx1+Blue LED. (b) Western blot of Trx1, NLRP3, and TXNIP proteins in the following four groups: control, Blue LED, shCtrl+Blue LED, and shTrx1+Blue LED. (c) Quantification of Trx1, NLRP3, and TXNIP protein expression. Data represent mean ± SD; *n* = 3 for each group. ^∗^*P* < 0.05 vs. control; ^∗∗^*P* < 0.01 vs. control; ^#^*P* < 0.05 vs. Blue LED; ^##^*P* < 0.01 vs. Blue LED; ^&^*P* < 0.05 vs. tBHQ; ^&&^*P* < 0.01 vs. tBHQ.

The effect of pretreatment with shTrx1 was evaluated on the expression of TXNIP at the mRNA (Figure 9(a)) and protein (Figures 9(b) and 9(c)) levels. In the nucleus, the expression of TXNIP was decreased in the Blue LED group compared with the control group. The expression was further decreased in the shTrx1+Blue LED group compared with the Blue LED and shCtrl+Blue LED groups. In contrast, the cytoplastic TXNIP expression was significantly increased in the Blue LED group compared with the control group. It was further increased in the shTrx1+Blue LED group compared with the Blue LED and shCtrl+Blue LED group. Besides, the Trx1 expression was decreased compared with the control group, whereas the NLRP3 expression was increased in the Blue LED group. However, compared with the Blue LED and shCtrl groups, the expression of Trx1 in the shTrx1+Blue LED group was significantly decreased, whereas the NLRP3 expression was significantly increased. The results showed that decreased Trx1 could significantly increase the expression of NLRP3 inflammasome. Furthermore, these results confirmed the translocation of TXNIP from the cytoplasm to the nucleus after Blue LED irradiation, which was promoted by the Trx1 knockdown in mouse retinas.

### 3.10. Effect of Nrf2 on TXNIP and NLRP3 in the Retina

**Figure 10:**
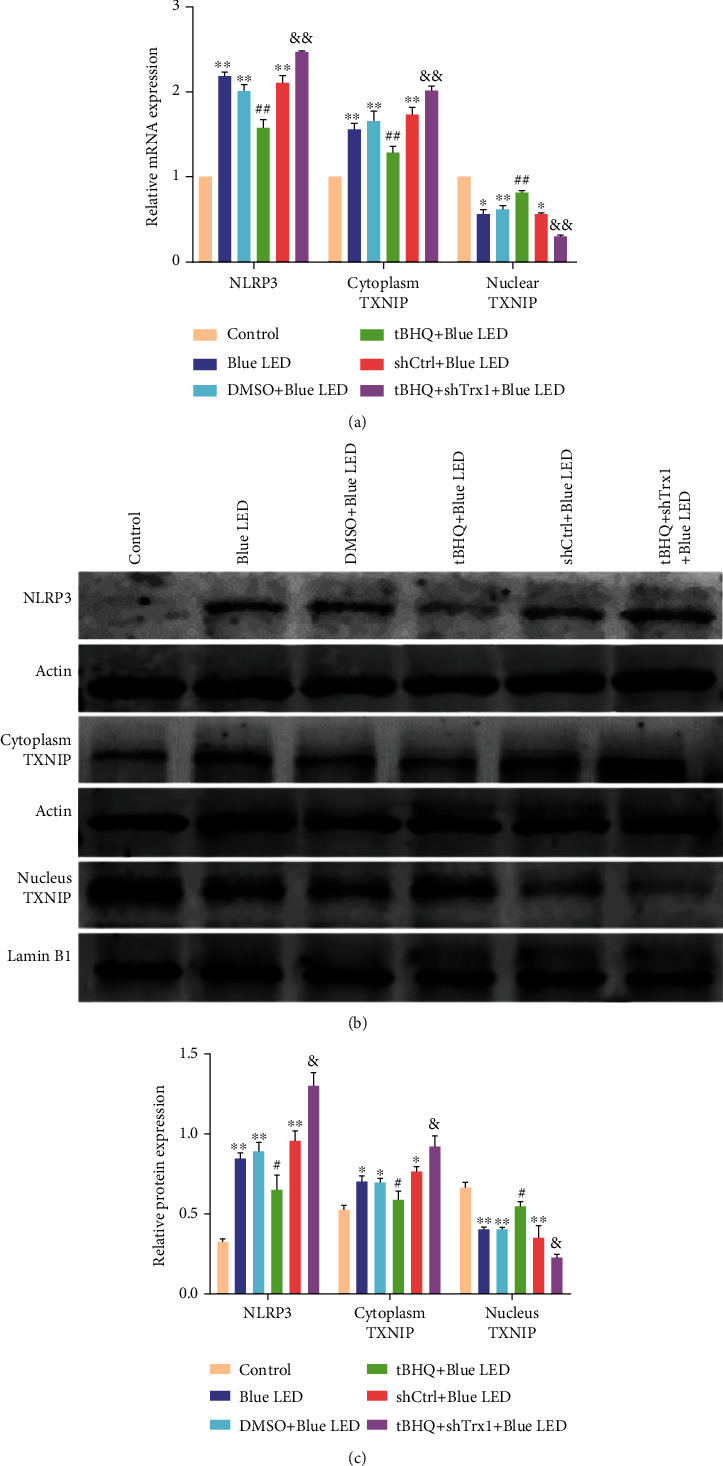
Effect of Nrf2 on TXNIP and NLRP3 in the retina. (a) RT-PCR analysis of mRNA in the following six groups: control, Blue LED, DMSO+Blue LED, tBHQ+Blue LED, shCtrl+Blue LED, and tBHQ+shTrx1+Blue LED. (b) Western blot of NLRP3 and TXNIP proteins in the following six groups: control, Blue LED, DMSO+Blue LED, tBHQ+Blue LED, shCtrl+Blue LED, and tBHQ+shTrx1+Blue LED. (c) Quantification of NLRP3 and TXNIP protein expression. Data represent mean ± SD; *n* = 3 for each group. ^∗^*P* < 0.05 vs. control; ^∗∗^*P* < 0.01 vs. control; ^#^*P* < 0.05 vs. Blue LED; ^#^*P* < 0.05 vs. Blue LED; ^##^*P* < 0.01 vs. Blue LED; ^&^*P* < 0.05 vs. Blue LED; ^&&^*P* < 0.01 vs. tBHQ+Blue LED.

A previous study had demonstrated that Nrf2 inhibited TXNIP in the oxidative stress cell model. Considering the present study findings of tBHQ treatment mediated upregulated Nrf2 and inhibited TXNIP transcription in mouse retinas, we examined the effects of Nrf2 and Trx1 on the inflammation-induced signaling cascade of TXNIP in vivo using qRT-PCR (Figure 10(a)) and western blot (Figures 10(b) and 10(c)). In the nucleus, compared with the control group, the expression of TXNIP in the Blue LED group was decreased, while the TXNIP expression was increased in the tBHQ+Blue LED group compared with the Blue LED and DMSO+Blue LED groups. Also, the tBHQ+shTrx1+Blue LED group showed that the expression of TXNIP further decreased compared with the tBHQ+Blue LED group. In addition, at the cytoplasm levels, compared with the control group, the TXNIP expression in the Blue LED group was increased, and the TXNIP expression was decreased in the tBHQ+Blue LED group compared to the Blue LED group. Meanwhile, the tBHQ+shTrx1+Blue LED group showed further added TXNIP expression than the tBHQ+Blue LED group. Furthermore, the expression of NLRP3 inflammasome changed synchronously with the cytoplasmic TXNIP. Nrf2 negatively regulated TXNIP transfer from the nucleus to the cytoplasm after Blue LED exposure, and Trx1 knockout could largely accelerate this effect.

### 3.11. Retinal Function Assessed by ERG and Pathological Changes of the Retina

**Figure 11:**
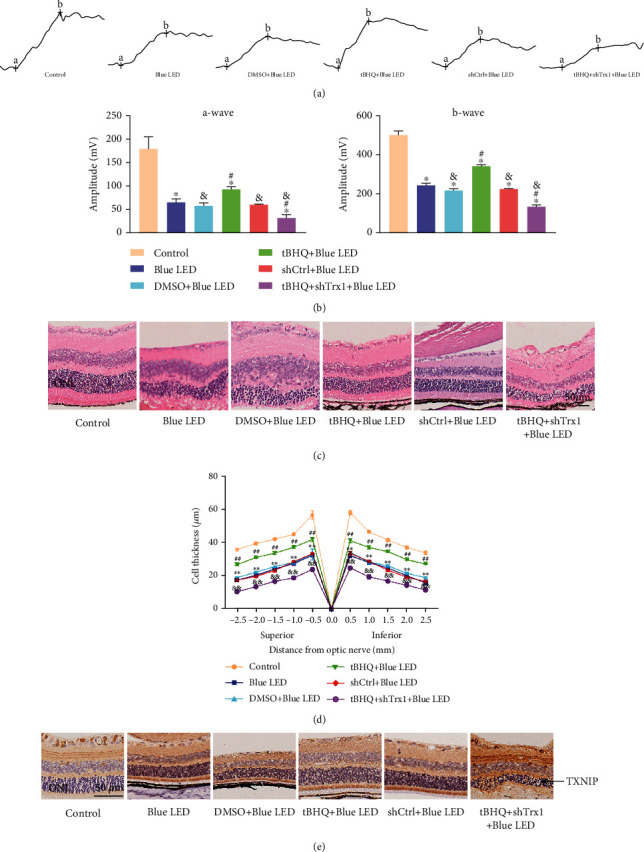
Effects of Nrf2 and Trx1 on retinal function and pathological structure. (a) Typical waveform of ERG. (b) Systematic analysis of the a-wave and b-wave of ERG. (c) ONL thickness. Magnification: ×500, scale bar: 50 *μ*m. (d) The statistical analysis of the ONL thickness. (e) TXNIP expression was examined by immunohistochemistry. Magnification: ×500, scale bar: 50 *μ*m. Data represent mean ± SD; the error bars represent the standard deviation of measurements for 7 separate sample (*n* = 7). ^∗^*P* < 0.05 vs. control; ^∗∗^*P* < 0.01 vs. control; ^#^*P* < 0.05 vs. Blue LED; ^##^*P* < 0.01 vs. Blue LED; ^&^*P* < 0.05 vs. Blue LED; ^&&^*P* < 0.01 vs. tBHQ+Blue LED.

For functional analysis, full-field ERG responses of tBHQ- or shTrx1-injected mice were compared 5 days after blue light exposure with control. Based on the ERG (Figures 11(a) and 11(b)), the amplitudes of a- and b-waves reduced in the Blue LED, DMSO+Blue LED, and ShCtrl+Blue LED groups compared to the control group. However, compared with Blue LED, the difference between the DMSO+Blue LED and ShCtrl+Blue LED groups was not statistically significant (*P* > 0.05). The tBHQ+Blue LED group had significantly higher amplitudes compared to Blue LED. Meanwhile, a- and b-wave amplitudes were significantly reduced in the tBHQ+shTrx1+Blue LED group than in the tBHQ+Blue LED group. The results confirmed that Nrf2 and Trx1 had protective effects on retinal function.

We evaluated the pathological structure of the retina using H&E and immunohistochemistry to investigate whether Nrf2 and Trx1 had protective effects on Blue LED-induced retinal injury. Based on the H&E assay (Figures 11(c) and 11(d)), we found that Blue LED caused structural damage and thinning of the ONL layer in the mice retina. However, the retinal morphology and structure disruption were restored in the tBHQ+Blue LED group compared to the Blue LED group. The thickness of ONL was reduced, and the morphology was disordered in the tBHQ+shTrx1+Blue LED group compared to the tBHQ+Blue LED group. Meanwhile, the TXNIP expression in the retina of mice exposed to LED was higher than in the control group (Figure 11(e)). Nrf2 treatment significantly reduced TXNIP expression compared with the mice exposed to the Blue LED, while knocking down Trx1 promoted an increased expression of TXNIP expression. Studies have confirmed that Nrf2 and Trx1 can save the damage to retina structure and morphology to a certain extent by maintaining the thickness of ONL.

## 4. Discussion

Previous studies have shown that inflammation is an important part of the development and progression of AMD [14]. The production of the NLRP3 inflammatory body activates caspase-1, induces pro-IL-1*β* and pro-IL-18 dissolution mediated inflammation, and aggravates retinal injury [3]. However, inflammatory corpuscles and the thioredoxin system have many activation pathways. TXNIP and Trx1 exist in the form of a complex [15]. TXNIP can further aggravate the effect of inflammation by reversing the inhibition of inflammation mediated by Trx1 [16]. Trx1 and TXNIP are ubiquitous in ARPE-19 cells [17]. Trx1 inhibits inflammation and is an inflammatory molecule in the extracellular and intracellular environment [18]. Some studies have confirmed that Trx1 combines with TXNIP in a resting state to form the complex. As a result, the inflammatory body cannot bind to TXNIP, rendering NLRP3 in an inactive form. Under oxidative stress, upon the dissociation of the Trx1/TXNIP complex, free TXNIP activates the NLRP3 inflammasome [19].

In the present study, we validated the association between Nrf2, Trx1/TXNIP, and NLRP3 inflammasomes in oxidative cell stress and light damage stress to the mice retina. Oxidative stress is one of the most important causes of early AMD [20]. The degeneration of ARPE-19 cells is a key to the pathogenesis of AMD [21]. H_2_O_2_ could induce ROS production in ARPE-19 cells, which are in oxidative stress [22]. H_2_O_2_ is one of the most common interventions for establishing oxidative stress cell models, which can mimic the oxidative damage in AMD and induce cells to produce ROS, leading to oxidative damage [23]. Therefore, in this study, H_2_O_2_ was used to treat ARPE-19 cells to establish an *in vitro* model. Additionally, 100 *μ*M H_2_O_2_ intervention for 1 h successfully established the oxidative damage model of cells. Nrf2 inhibited the oxidative stimulation, alleviated the oxidative reaction of cells by regulating a series of antioxidant reactions, and kept the body in a state of homeostasis. These factors are important to maintain the oxidative balance of organisms [24]. Moreover, TXNIP could activate inflammatory factors in neurodegenerative diseases, similar to the results of our study [25, 26]. Our study found that H_2_O_2_ can increase the TXNIP level in the cytoplasm and decrease it in the nucleus. The pretreatment with 30 *μ*M tBHQ to activate Nrf2 significantly decreased ROS production, accelerated cell healing, increased cell migration, and alleviated the oxidative damage of mitochondria. However, knocking down Trx1 increased ROS production, delayed cell healing, reduced cell migration, and aggravated mitochondrial damage [27, 28]. The results were consistent with previous findings, indicating that Nrf2 and Trx1 have antioxidant effects in ARPE-19 cells [29, 30].

Blue light is a definite risk factor for AMD. Blue light irradiation increases the concentration of free radicals in the eyes and destroys the original physiological balance. The oxidative stress model of AMD induced by LED is a classical retinal injury model related to this specific wavelength [31]. Therefore, researchers have started using the retinal damage model induced by Blue LED to study the pathophysiological changes of AMD and evaluate the effect of new therapeutic drugs. Exposure to LED at 460 nm causes histopathological and functional changes in the animal retina [32]. Therefore, after blue LED (3000 lux, 460 nm) irradiation, ERG and H&E were used to evaluate the influence of retinal function and retinal structure, respectively. The a- and b-wave amplitudes of ERG were significantly decreased, many photoreceptors were lost, retinal cells underwent pyknosis, and the thickness of ONL was decreased in mice exposed to Blue LED radiation. Of note, these results were consistent with previous studies [33]. During *in vivo* experiments, injection of 2 *μ*L of tBHQ and shTrx1 was administered into the vitreous cavity of mice, followed by exposure to the blue light. The tBHQ-activated Nrf2 significantly increased the a- and b-wave amplitudes in ERG, while the thickness of the ONL layer was increased compared with the Blue LED group. Meanwhile, Trx1 knockout further reduced the a- and b-wave amplitudes in ERG, and the thickness of the ONL layer was reduced compared with the tBHQ+Blue LED group.

Some studies have confirmed that Trx1 has a protective role in the nervous system [34]. However, no studies have shown a direct association between the Trx1/TXNIP complex with NLRP3 inflammasomes at cellular and animal levels for AMD pathogenesis. Thus, we knocked down Trx1 based on activation of Nrf2 and observed the changes in TXNIP expression in the nucleus and cytoplasm for NLRP3. Decreasing Trx1 significantly increased the cytoplasmic TXNIP and decreased nuclear TXNIP, thereby increasing the expression of the NLRP3 inflammasome. This phenomenon resulted from a decrease in Trx1, which could separate TXNIP from the nucleus and transfer it to the cytoplasm to activate the NLRP3 inflammasome. The present study confirmed the association between Trx1, TXNIP, and NLRP3 inflammasomes in oxidized-damaged ARPE-19 cells and the retina of light-damaged mice.

Previous *in vitro* studies have found that tBHQ activates Trx1 through the Nrf2 pathway, and Trx1 is regulated by Nrf2 [35]. At the protein and gene levels, the Trx1 and Nrf2 expression in oxidative damage of ARPE-19 cells and retina of light-damaged mice was less than in the control group. After activation of Nrf2 by tBHQ, the protein and gene expression of Nrf2 increased, while NLRP3 and its downstream caspase-1, IL-18, and IL-1*β* also increased. Nrf2 could inhibit the activation of NLRP3 inflammasomes. This concurs with our results showing anti-inflammatory effects of Nrf2 and Trx1 by immunofluorescence. We tested the levels of light-damaged mouse retina and oxidative damaged ARPE-19 cells to assess the effect of Nrf2 on inflammation related to the Trx1/TXNIP complex. Nonetheless, Nrf2 activation reduced the cytoplasmic TXNIP expression and alleviated NLRP3 inflammatory response. The nuclear expression of TXNIP increased due to Trx1 binding induced by Nrf2 activation. For further validation, we knocked down Trx1 based on tBHQ activation. The TXNIP metastasis was more obvious than Nrf2 activation, suggesting that Nrf2 regulated inflammation via the Trx1/TXNIP complex.

## 5. Conclusions

Cytoplasmic TXNIP activated inflammatory factors in the retina of blue LED-induced light injury mice and H_2_O_2_-induced oxidative stress cells. This pathway was achieved by regulating the Trx1/TXNIP complex by Nrf2. These findings may provide new ideas for the future treatment of AMD.

## Figures and Tables

**Table 1:** Primer sequences used for PCR.

Gene symbols	Forward 5′–3′	Reverse 5′–3′
Nrf2 (cell)	TTAAACCTGCGGACACTGTTG	GTATCCCAAGGCGTTCTTGTT

TXNIP	GGTCTTTAACGACCCTGAAAAGG	ACACGAGTAACTTCACACACCT

Trx1	GTGAAGCAGATCGAGAGCAAG	CGTGGCTGAGAAGTCAACTACTA

NLRP3	GATCTTCGCTGCGATCAACAG	CGTGCATTATCTGAACCCCAC

IL-1*β*	CCTCTCTCTAATCAGCCCTCTG	GAGGACCTGGGAGTAGATGAG

IL-18	TCTTCATTGACCAAGGAAATCGG	TCCGGGGTGCATTATCTCTAC

Caspase-1	TTTCCGCAAGGTTCGATTTTCA	GGCATCTGCGCTCTACCATC

GAPDH	GGAGCGAGATCCCTCCAAAAT	GGCTGTTGTCATACTTCTCATGG

18S	CATGTACGTTGCTATCCAGGC	CTCCTTAATGTCACGCACGAT

Nrf2 (mice)	CGGGGACGCAGTGATGTATG	TGTGTAGCTGAAGGTTCGGTTA

TXNIP	GGCCGGACGGGTAATAGTG	AGCGCAAGTAGTCCAAAGTCT

Trx1	TTCCCTCACCTCTAAGACCCT	GTCAGGCTCTTCCACTCATCTAT

NLRP3	TGACCCTTATGACCAGTCCTTT	GTCAGGCTCTTCCACTCATCTAT

IL-1*β*	GAAATGCCACCTTTTGACAGTG	TGGATGCTCTCATCAGGACAG

IL-18	CTGTGGACATATTCTGCAAGGG	GCATGTACCACTGGACATCAGAT

Caspase-1	ATTACCCGCCCGAGAAAGG	CATGAGTGTGGCTAGATCCAAG

GAPDH	AGGTCGGTGTGAACGGATTTG	GGGGTCGTTGATGGCAAC

18S	GCTGGAGGACTCATGTTCAAC	GCACCACATCGGTAGGTCTTAAA

## Data Availability

Data used to support the findings of this study are available from the corresponding author upon request.

## References

[1] Schnabolk G. (2019). Systemic inflammatory disease and AMD comorbidity. *Advances in Experimental Medicine and Biology*.

[2] Yerramothu P., Vijay A. K., Willcox M. D. P. (2018). Inflammasomes, the eye and anti-inflammasome therapy. *Eye (London, England)*.

[3] Gao Z., Li Q., Zhang Y., Gao X., Li H., Yuan Z. (2020). Ripasudil alleviated the inflammation of RPE cells by targeting the miR-136-5p/ROCK/NLRP3 pathway. *BMC Ophthalmology*.

[4] Huang P., Liu W., Chen J. (2020). TRIM31 inhibits NLRP3 inflammasome and pyroptosis of retinal pigment epithelial cells through ubiquitination of NLRP3. *Cell Biology International*.

[5] Ren C., Liu Q., Wei Q. (2017). Circulating miRNAs as potential biomarkers of age-related macular degeneration. *Cellular Physiology and Biochemistry : International Journal of Experimental Cellular Physiology, Biochemistry, and Pharmacology*.

[6] Pan M., Zhang F., Qu K., Liu C., Zhang J. (2022). TXNIP: a double-edged sword in disease and therapeutic outlook. *Oxidative Medicine and Cellular Longevity*.

[7] Chen W., Zhao M., Zhao S. (2017). Activation of the TXNIP/NLRP3 inflammasome pathway contributes to inflammation in diabetic retinopathy: a novel inhibitory effect of minocycline. *Inflammation research : official journal of the European Histamine Research Society [et al]*.

[8] Hou Y., Wang Y., He Q. (2018). Nrf2 inhibits NLRP3 inflammasome activation through regulating Trx1/TXNIP complex in cerebral ischemia reperfusion injury. *Behavioural brain research*.

[9] Holubiec M. I., Galeano P., Romero J. I., Hanschmann E. M., Lillig C. H., Capani F. (2020). Thioredoxin 1 Plays a Protective Role in Retinas Exposed to Perinatal Hypoxia- Ischemia. *Neuroscience*.

[10] Sugano E., Isago H., Murayama N., Tamai M., Tomita H. (2013). Different anti-oxidant effects of thioredoxin 1 and thioredoxin 2 in retinal epithelial cells. *Cell Structure and Function*.

[11] (2015). Erratum: Cytotoxicity and physicochemical characterization of iron-manganese-doped sulfated zirconia nanoparticles [Corrigendum]. *International journal of nanomedicine*.

[12] Ji Y. J., Wang H. L., Yin B. L., Ren X. Y. (2020). Down-regulation of DJ-1 Augments Neuroinflammation via Nrf2/Trx1/NLRP3 Axis in MPTP-induced Parkinson's Disease Mouse Model. *Neuroscience*.

[13] Mao K., Shu W., Qiu Q., Gu Q., Wu X. (2017). Salvianolic acid A protects retinal pigment epithelium from OX-LDL-induced inflammation in an age-related macular degeneration model. *Discovery Medicine*.

[14] Telander D. G. (2011). Inflammation and age-related macular degeneration (AMD). *Seminars in Ophthalmology*.

[15] Wang C. Y., Xu Y., Wang X., Guo C., Wang T., Wang Z. Y. (2019). Dl-3-n-Butylphthalide inhibits NLRP3 inflammasome and mitigates Alzheimer’s-like pathology via Nrf2-TXNIP-TrX axis. *Antioxidants & Redox Signaling*.

[16] Liu H., Guo W., Guo H. (2020). Bakuchiol attenuates oxidative stress and neuron damage by regulating Trx1/TXNIP and the phosphorylation of AMPK after subarachnoid hemorrhage in mice. *Frontiers in Pharmacology*.

[17] Sreekumar P. G., Ding Y., Ryan S. J., Kannan R., Hinton D. R. (2009). Regulation of thioredoxin by ceramide in retinal pigment epithelial cells. *Experimental Eye Research*.

[18] Shi Y., Jin Y., Liu F. (2021). Ceramide induces the apoptosis of non-small cell lung cancer cells through the Txnip/Trx1 complex. *International journal of molecular medicine*.

[19] Xu L., Lin X., Guan M., Zeng Y., Liu Y. (2019). Verapamil attenuated prediabetic neuropathy in high-fat diet-fed mice through inhibiting TXNIP-mediated apoptosis and inflammation. *Oxidative Medicine and Cellular Longevity*.

[20] Wiktorowska-Owczarek A., Nowak J. Z. (2010). Pathogenesis and prophylaxis of AMD: focus on oxidative stress and antioxidants. *Postepy higieny i medycyny doswiadczalnej (Online)*.

[21] Brown E. E., DeWeerd A. J., Ildefonso C. J., Lewin A. S., Ash J. D. (2019). Mitochondrial oxidative stress in the retinal pigment epithelium (RPE) led to metabolic dysfunction in both the RPE and retinal photoreceptors. *Redox biology*.

[22] Keeling E., Chatelet D. S., Johnston D. A. (2019). Oxidative stress and dysfunctional intracellular traffic linked to an unhealthy diet results in impaired cargo transport in the retinal pigment epithelium (RPE). *Molecular Nutrition & Food Research*.

[23] Tong Y., Wang S. (2020). Not all stressors are equal: mechanism of stressors on RPE cell degeneration. *Frontiers in Cell and Developmental Biology*.

[24] Du Y., You L., Ni B. (2020). Phillyrin Mitigates Apoptosis and Oxidative Stress in Hydrogen Peroxide- Treated RPE Cells through Activation of the Nrf2 Signaling Pathway. *Oxidative Medicine and Cellular Longevity*.

[25] Li L., Ismael S., Nasoohi S. (2019). Thioredoxin-interacting protein (TXNIP) associated NLRP3 inflammasome activation in human Alzheimer’s disease brain. *Journal of Alzheimer's disease : JAD*.

[26] Hu L., Zhang H., Wang B., Ao Q., He Z. (2020). MicroRNA-152 attenuates neuroinflammation in intracerebral hemorrhage by inhibiting thioredoxin interacting protein (TXNIP)-mediated NLRP3 inflammasome activation. *International Immunopharmacology*.

[27] Yumnamcha T., Devi T. S., Singh L. P. (2019). Auranofin mediates mitochondrial dysregulation and inflammatory cell death in human retinal pigment epithelial cells: implications of retinal neurodegenerative diseases. *Frontiers in Neuroscience*.

[28] Hanus J., Kolkin A., Chimienti J., Botsay S., Wang S. (2015). 4-Acetoxyphenol prevents RPE oxidative stress-induced necrosis by functioning as an NRF2 stabilizer. *Investigative Ophthalmology & Visual Science*.

[29] Felszeghy S., Viiri J., Paterno J. J. (2019). Loss of *NRF-*2 and *PGC-*1 *α* genes leads to retinal pigment epithelium damage resembling dry age-related macular degeneration. *Redox Biology*.

[30] Liu X., Ward K., Xavier C. (2016). The novel triterpenoid RTA 408 protects human retinal pigment epithelial cells against H_2_O_2_-induced cell injury via NF-E2-related factor 2 (Nrf2) activation. *Redox Biology*.

[31] Krigel A., Berdugo M., Picard E. (2016). Light-induced retinal damage using different light sources, protocols and rat strains reveals LED phototoxicity. *Neuroscience*.

[32] Kim G. H., Kim H. I., Paik S. S., Jung S. W., Kang S., Kim I. B. (2016). Functional and morphological evaluation of blue light-emitting diode-induced retinal degeneration in mice. *Graefe's archive for clinical and experimental ophthalmology = Albrecht von Graefes Archiv fur klinische und experimentelle Ophthalmologie*.

[33] Wei Q., Liang X., Peng Y. (2018). 17*β*-estradiol ameliorates oxidative stress and blue light-emitting diode-induced retinal degeneration by decreasing apoptosis and enhancing autophagy. *Drug design, development and therapy*.

[34] Munemasa Y., Kim S. H., Ahn J. H., Kwong J. M. K., Caprioli J., Piri N. (2008). Protective effect of thioredoxins 1 and 2 in retinal ganglion cells after optic nerve transection and oxidative stress. *Investigative Ophthalmology & Visual Science*.

[35] Liu X., Zhang X., Ding Y. (2017). Nuclear factor E2-related factor-2 negatively regulates NLRP3 inflammasome activity by inhibiting reactive oxygen species-induced NLRP3 priming. *Antioxidants & Redox Signaling*.

